# Using adaptive optics to assess hyporeflective clump speed and size in age-related macular degeneration in the PINNACLE Study. (PINNACLE Study Report 6)

**DOI:** 10.1038/s41433-025-03951-7

**Published:** 2025-09-04

**Authors:** Christopher Holmes, Dimitrios Kazantzis, Syed Ahmer Raza, Naomi Wijesingha, Thomas RP Taylor, Ahmed Hagag, Sophie Riedl, Julia Mai, Daniel Rueckert, Hrvoje Bogunović, Hendrik Scholl, Ursula Schmidt-Erfurth, Lars Fritsche, Andrew Lotery, Sobha Sivaprasad

**Affiliations:** 1 NIHR Moorfields Clinical Research Facility, Moorfields Eye Hospital NHS Foundation Trust, London, UK; 2 University of Southampton, Faculty of Medicine, Southampton, UK; 3 University College London Institute of Ophthalmology, London, UK; 4 Boehringer Ingelheim Limited, Bracknell, UK; 5 Medical University of Vienna, Vienna, Austria; 6 Imperial College London, London, UK; 7 Institute of Molecular and Clinical Ophthalmology Basel, Basel, Switzerland; 8 Department of Ophthalmology, University of Basel, Basel, Switzerland; 9 University of Michigan, Ann Arbor, MI USA

**Keywords:** Macular degeneration, Prognostic markers

## Abstract

**Background/Objectives:**

Hyporeflective clumps (HRC) are a common finding in adaptive optics ophthalmoscopy (AOO) of age-related macular degeneration (AMD). They appear on optical coherence tomography (OCT) as hyperreflective foci (HRF) or abutting the retinal pigment epithelium (RPE) layer as RPE thickening. The cellular origin of HRF is debated between migrated RPE cells and mononuclear phagocytes (MP). Microglial cells are MP known to migrate at 0.02 µm/s, but RPE migration speed is unknown. Phenotyping HRCs by migration speed and size may improve our understanding of HRFs.

**Methods:**

Patients with non-neovascular AMD were imaged with the RTX1 retinal camera (Imagine Eyes, Orsey, France). Pairs of AOO images taken 1–3 h apart were centred on areas with multiple HRCs and compared to identify mobile HRCs. Macular OCT scans were performed immediately after initial AOO.

**Results:**

A total of 21 pairs of images from 14 eyes of 12 patients were of adequate quality to assess HRCs. There were 411 measurable HRCs, with a mean diameter of 15.9 ± 6.0 µm. The HRCs were larger in images of atrophy (*p* < 0.001). Within the timeframe assessed, most HRCs remained static, but mobile HRCs were not uncommon and migrated up to 0.015 µm/s. HRFs on OCT corresponding to mobile HRCs on AOO appeared adjacent to the RPE or in the interdigitation zone.

**Conclusion:**

AOO can detect HRC movement in AMD in images captured a mean of 105.5 min apart. HRC size and movement speed are consistent with microglial cells, but may also represent RPE cells. HRCs appear larger in images of atrophy.

## Introduction

Age-related macular degeneration (AMD) is a complex disease predominantly affecting the macula. It results in progressive central vision loss and is the leading cause of blindness in older individuals in developed countries. AMD is categorised using the Beckman classification [1], but developments in optical coherence tomography (OCT) imaging have enabled further identification of biomarkers, including hyper-reflective foci (HRF) [2]. HRF in AMD appears as bright dots or roundish lesions within retinal layers on OCT, but alongside retinal pigment epithelium (RPE) thickening, they appear deeply hypo-reflective on flood illumination adaptive optics ophthalmoscopy (AOO) and are known as hypo-reflective clumps (HRCs) [3]. AOO is an imaging technique that compensates for optical aberrations using a deformable mirror or computed algorithms, informed by wavefront measurement. This technology enables high-resolution imaging of the living retina and may provide new insights into the cellular mechanisms underlying AMD.

The cellular origin of HRFs remains uncertain, but proposed theories include migrated RPE [2, 4] cells or melanosome-containing mononuclear phagocytes (MP) [5–7]. MPs are a group of cells including monocytes, resident and monocyte-derived inflammatory macrophages, and microglial cells [8].

Microglial activation in AMD is thought to result in outer retinal accumulation of melanosome-laden macrophages [7]. Both migrated RPE cells and macrophages may relocate over time. To our knowledge, the speed of movement of RPE cells has not been described in humans, but microglia can migrate 0.02 µm/s in healthy retinas and have been shown to move faster (2.37 µm/s) when inflamed [9]. HRCs are also known to migrate [3, 10], but their speed of movement over short time periods has not been determined. With AOO, we can observe HRC morphology and behaviour in vivo, which could create new cell phenotypes and imaging biomarkers, and may have important implications for the mechanism of progression and management of non-neovascular AMD.

This study aimed to measure the size and speed of movement of HRCs to provide a method of phenotyping HRCs and advance our knowledge of in vivo cellular behaviour in the retina. To this end, we assessed repeat AOO images within hours to determine the speed of an HRC’s movement.

## Methods

The PINNACLE study is an international multi-centre observational study aiming to create prognostic models of intermediate AMD. The study adhered to the principles of Good Clinical Practice in accordance with the declaration of Helsinki and received approval from the East Midlands—Leicester Central Research Ethics Committee (ref. 19/EM/0163).

Patients with intermediate AMD in one or both eyes were recruited for the trial based on a pre-defined protocol [11]. Informed consent was obtained from all patients. Included eyes could have incomplete Retinal and Outer Retinal Atrophy (iRORA), but both complete Retinal and Outer Retinal Atrophy (cRORA) and neovascular AMD were exclusion criteria. Patients who developed neovascular AMD exited the study, but those who developed cRORA after enrolment continued in the trial, some of whom were included in this sub-study. The patients included in this sub-study were a group of consecutively assessed patients at pre-defined time points between November 2022 and January 2023 in line with the trial protocol. They were included if they agreed to repeat AOO imaging and were found to have HRCs on AOO. Both cRORA and intermediate AMD patients were included, as HRCs were detectable in all patients.

### Image acquisition

For the main PINNACLE study, full clinical imaging with pupil dilation at set time-points was performed in accordance with the trial protocol [11]. For the purposes of this study, only infra-red reflectance SLO, spectral domain OCT (Spectralis HRA + OCT; Heidelberg Engineering, Heidelberg, Germany), and AOO (rtx1 AO Retinal Camera, Imagine Eyes, Orsay, France) imaging were assessed.

**Fig. 1:**
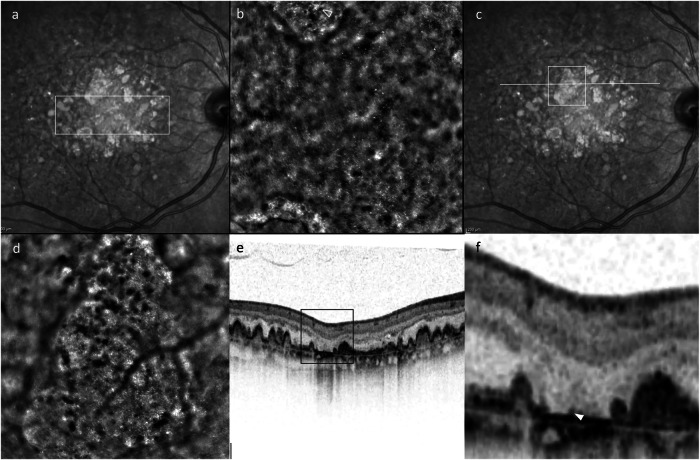
Selection of hyporeflective clump (HRC) containing lesions, and comparison with optical coherence tomography (OCT) B scans to identify corresponding hyperreflective foci (HRF). Near infra-red scanning laser ophthalmoscopy image **a** of a patient with late non-neovascular age-related macular degeneration, displaying the area assessed with 5 overlapping adaptive optics ophthalmoscopy (AOO) images. AOO images **b** were assessed to identify HRC containing lesions where the HRC margins were clear and well defined (e.g., empty white triangle). Further AOO images centred on the area of interest (white square, **c**) were captured (**d**). After a mobile HRC was identified (white arrow), the closest B scan slice (white line, **c**), was reviewed with inverted colours (**e**) and magnified **f** to identify the corresponding HRF. Also visible in **d** are multiple small (2.3–10 µm diameter), medium (10–25 µm diameter), and large (>25 µm diameter) HRCs.

Each patient in this study underwent AOO, including 5 horizontally overlapping images covering an area of 12^o^ horizontally by 4^o^ vertically, centred on the patient’s point of fixation. If present, one or more additional images were acquired of areas within the arcades with high concentrations of HRCs with clear and well-defined margins. These areas were identified if present on the 5 overlapping images (Fig. 1a), or review of OCT to show areas with HRF or RPE thickening, which was defined as a localised increase in RPE thickness compared to normal RPE within the same eye. The corresponding areas were imaged with AOO using landmarks such as retinal vessels as a guide. Focal depth was selected to show HRCs clearly at the level of the cone mosaic. Immediately after AOO, high-density 20° by 20° macular spectral domain OCT scans were performed, with 193 B-scans per volume and simultaneous 30° by 30° infra-red SLO imaging.

At a single further timepoint, aimed 1–3 h after the initial AOO imaging, repeat AOO was performed, selectively targeting one or more areas identified in initial imaging with high concentrations of HRCs. Both AOO images in each series were targeted at the same focal depth.

### Image processing and interpretation

Individual AOO images were assessed to minimise image manipulation. To enable alignment of corresponding location-matched images, compensation for image rotation was performed with the cloud-based image-editing programme PIXLR (available at https://pixlr.com/). Alignment was considered complete once maximum concordance between the two overlain images was achieved. The overlaid images were then compared to identify differences in HRCs between the images (supplementary video 1), and HRCs that did not align were considered candidates for movement. Within each image, the distance between landmark features (such as retinal vessels or margins of atrophy) was measured with AOdetect^TM^ software (ImagineEyes). The wall-detect function, which was designed to measure vessel lumens, was used to measure distance between two image landmarks in µm, and the mean of three measurements taken as the distance. Landmarks were chosen to be as far apart as possible to minimise human error when measuring the distance. Axial length data was not available as this was not collected as art of the Pinnacle study, but a default axial length of 24 mm was used to convert pixel size to µm using AOdetect^TM^. These measurements were used to calibrate pixel-based measurements taken in ImageJ (version 1.53t) [12]. When measuring HRC diameter, the clearest image in the series was used, and all HRCs were assessed where their borders were well defined and not overlapping with other HRCs. Where HRCs were circular, one measurement was taken, and where they were amoeboid in shape, the mean of the longest and shortest axis was taken. Measurements of HRC movement were used only where cell origin and destination were considered highly likely. Likelihood was evaluated by assessing HRC shape, location, size, and reflectivity, as well as adjacent features such as possible confusion with other mobile HRCs. Using PIXLR, annotations were made on the baseline image as straight lines between the centres of HRC origin and destination. When HRCs were circular, the HRC centre was marked as origin or destination, and when HRCs appeared non-circular, annotations were made from the mid-point of the HRC’s longest axis. The length of the annotation line was then measured with ImageJ. Two authors separately measured the distance, and the inter-rater agreement was measured using the kappa coefficient (*k*). The agreement was less than chance if *k* < 0, there was slight agreement if 0.01 < *k* < 0.2, fair agreement if 0.21 < *k* < 0.4, moderate agreement if 0.41 < *k* < 0.6, substantial agreement if 0.61 < *k* < 0.8, and almost perfect agreement if 0.81 < *k* < 0.99. Measurements of distance were divided by time to give HRC speed in µm/s, and only the greatest HRC speed was used for each image. The identified mobile HRC was correlated with HRF or RPE thickening on OCT against the first AOO image in the series, using landmarks such as retinal vessels as a reference (Fig. 1). Two raters agreed on the most likely HRC, but inter-rater agreement was not assessed due to the small sample size.

### Statistical analysis

Given the small number of patients, the results are presented in a descriptive manner, with averages used representing the mean ± standard deviation for HRC size, or with the range for HRC movement. Statistical analysis of measurements of HRC diameter was performed with independent samples *T*-tests to compare between groups. The inter-rater agreement was assessed using the kappa coefficient (*k*). Statistical tests were performed using Jamovi software (version 1.6.23) and Stata/BE V.18.0 (STATA Corp., College Station, TX, USA).

## Results

### The size of HRCs

**Fig. 2:**
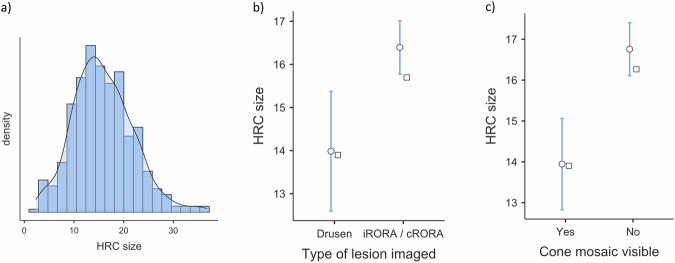
Descriptive plots of hyporeflective clump size distribution. The histogram/density plot **a** of HRC size shows a normal distribution. Grouping HRC size by type of lesion imaged **b** showed significantly larger HRCs over complete retinal pigment epithelium and outer retina atrophy (cRORA) compared to drusen (*p* < 0.001), and similarly, grouping HRC size by whether the cone mosaic was mostly intact **c**, showed that a mostly intact cone mosaic on the AOO image was associated with smaller HRCs (*p* < 0.001). The ○ represents mean, the □ median, and the bars the 95% confidence interval.

A total of 21 non-overlapping image series from 14 eyes in 12 patients were of adequate quality to assess HRCs' size. All 12 patients were white British, 4 were male and 8 female, and their mean age was 76.2 (range 65-86). HRCs appeared to frequently cluster together, with many instances of two or more presumed overlapping circular HRCs. This was not formally assessed, but HRC clustering made their number and size challenging to assess in many cases. Margins were identifiable and measured in 411 HRCs. Mean HRC size was 15.9 µm (±6.0 µm) across all images. Mean HRC size was similar across different patients and between eyes in the same patients (Table 1). HRC size showed a normal distribution (Fig. 2a) although there appeared to be smaller HRCs that were more common in some images. HRC measurements were compared between images containing iRORA or cRORA (overlying or up to 3^o^ from the lesion edge), and those that only contained medium (63–125 µm) and large (>125 µm) drusen, which were grouped together for analysis due to the small sample size. Mean HRC size appeared larger in images with iRORA or cRORA compared to those with just drusen (Fig. 2b, 16.6 μm vs 14.2 μm, *p* < 0.001). Similarly, areas with mostly intact cone mosaics (visible in >50% of the image) showed smaller HRCs (13.9 μm) than those where the cone mosaic was mostly absent (16.8 μm, *p* < 0.001). Correlation with OCT findings was challenging due to the differences in resolution and dimensionality between the two imaging modalities. HRCs around the level of the photoreceptors or RPE appeared to have the clearest margins in these images, but close clumping of HRCs in many cases made identification of the depth of individual HRCs challenging. HRFs on OCT were seen in all lesions where HRCs were observed on AOO, but many more individual HRCs were identifiable on AOO imaging than HRFs on OCT. OCT was, however, better at identifying individual HRFs within clumps, particularly on the inverting image signal.

**Table 1:** Mean HRC size by patient and eye, with corresponding pathology present in each AOO image.

Patient number	Eye	Number of HRCs measured	Mean HRC diameter (range) [µm]	Pathology imaged^a^	Cone mosaic visible^b^
1	Left	16	16.5 (5.6–20.9)	Large drusen	No
2	Right	18	13.8 (9.1–26.2)	Large drusen	Yes
3	Right	19	18.0 (3.2–34.8)	Medium drusen	Yes
4	Right	27	12.32 (3.1–26.4)	iRORA	Yes
5	Right	10	16.8 (10.6–24.0)	cRORA	No
5	Right	9	19.4 (9.5–27.1)	cRORA	No
5	Right	23	21.1 (13.0–34.0)	cRORA	No
5	Right	26	12.9 (4.0–28.4)	cRORA	No
6	Left	6	16.1 (11–24.3)	cRORA	No
6	Left	35	15.8 (9.8–23.0)	cRORA	No
7	Left	15	12.0 (7.7–18.3)	cRORA	No
8	Right	13	20.0 (15.7–26.1)	cRORA	No
9	Left	22	7.3 (2.3–14.0)	Medium drusen	Yes
10	Right	16	16.0 (7.9–28.7)	Medium drusen	No
11	Left	27	20.1 (9.8–30.4)	cRORA	No
11	Left	22	14.0 (6.8–22.0)	cRORA	No
11	Left	29	19.0 (11.7–27.1)	cRORA	No
11	Right	11	20.1 (14.5–37.7)	cRORA	No
12	Right	16	13.6 (7.4–18.8)	cRORA	Yes
12	Right	27	16.8 (9.4–24.9)	cRORA	No
12	Left	17	14.6 (5.4–20.9)	cRORA	No

^a^Most advanced pathology within the AOO image, determined by OCT and SLO findings.

^b^Cone mosaic visibility loss in >50% of the AOO image.

### The movement of HRCs

**Fig. 3:**
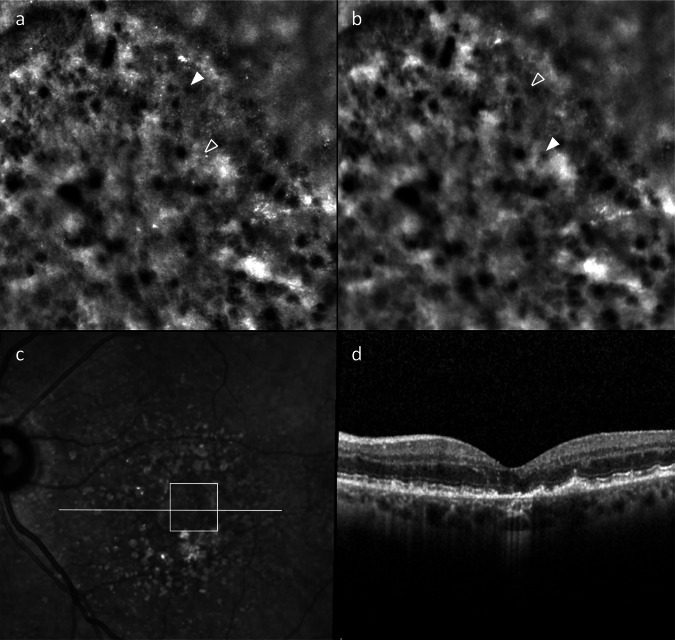
Adaptive optics ophthalmoscopy image series showing hyporeflective clump (HRC) migration within complete retinal pigment epithelium and outer retina atrophy (cRORA). AOO images focused at the level of the photoreceptors on the temporal edge of a cRORA lesion at baseline (**a**) and 136 min later (**b**). The cRORA appears as a relatively hyper-reflective area in the lower left half of both images without a visible cone mosaic. The transitional zone appears relatively hypo-reflective, possibly due to HRCs viewed through a partially visible cone mosaic. Marked with full white triangles are the origin and destination of the same HRC in both images, conspicuous by their absence in the matched image (empty triangles). Mobile HRCs are much more apparent in video format, as shown in supplementary video 1. Image **c**) shows the near infra-red scanning laser ophthalmoscopy image taken shortly after the baseline AOO, with the white square denoting the area captured in the AOO images. The white line shows the section captured in the optical coherence tomography (OCT) B scan (**d**). On the OCT B scan, HRFs can be seen just above the RPE within the cRORA, as well as in the EZ, Henle’s fibre layer, and the outer nuclear layer in the transitional zone. Multiple HRFs are visible elsewhere in the OCT B scan in various retinal layers.

18 images from 13 eyes of 11 patients were of adequate quality to assess HRCs in both images, at a mean time interval of 105.5 min (range 49-151). Detectable HRC movement between images was seen in every series assessed (Fig. 3, supplementary video 1), but clear origins and destinations of HRCs could only be identified in 15 image series from 10 eyes in 8 patients. Factors making origin and destination uncertain included image exposure, large distances moved, high concentrations of HRCs, multiple moving HRCs, changes in focal depth of the image or changes in depth of the HRC. No evidence of movement was seen in image series separated by 2 min or less. The maximum identifiable HRC movement from each image, the mobile HRC’s likely location on OCT, and the type of lesion imaged are recorded in Table 2. Identifying HRFs on OCT that corresponded to mobile HRCs was challenging due to clustering and unreliable visibility on OCT, but the HRFs that raters agreed corresponded to moving HRCs were all RPE adjacent, appearing as RPE thickening, or at the level of the photoreceptor inner or outer segments. The mean size of the mobile HRCs was 14.1 µm (range 7.9–20.8 µm) and was similar between iRORA/cRORA (14.4 µm, range 9.0–20.8 µm) and drusen patients (11.8 µm, range 7.9–15.7 µm). The majority of HRCs remained static within the timeframe of the imaging protocol, but mobile HRCs were found in every image. Mobile HRCs travelled a mean of 39.4 µm (range 6.9–82.7 µm), but assuming linear movement, they migrated up to 0.015 µm/s (mean fastest HRC 0.006 µm/s) with substantial inter-rater agreement (*k* = 0.65).

**Table 2:** Size and speed of the fastest-moving HRCs in each AOO image series, with suspected location and underlying AMD changes on OCT.

Patient number	Eye	Mobile HRC diameter (μm)	Time between images (min)	Speed (µm/s)	Retinal location	Lesion type
4	Right	9	49	0.0054	RPE	iRORA
5	Right	13.4	115	0.0044	RPE	cRORA
5	Right	17.9	110	0.0044	RPE	cRORA
5	Right	17	108	0.0068	RPE	cRORA
6	Left	9.8	86	0.0055	RPE	cRORA
7	Left	8.8	151	0.0078	RPE	cRORA
8	Right	15.7	72	0.0146	RPE	large drusen
10	Right	7.9	92	0.0075	IZ	medium drusen
11	Right	19.1	118	0.0022	RPE	cRORA
11	Left	14.1	136	0.0101	RPE	cRORA
11	Left	20.8	137	0.0008	RPE	cRORA
11	Left	14.6	131	0.0098	RPE	cRORA
12	Right	15.7	94	0.0029	RPE	cRORA
12	Right	12.7	101	0.0068	IZ	cRORA
12	Left	14.5	93	0.0055	RPE	cRORA

*RPE* retinal pigmented epithelium adjacent, *IZ* interdigitation zone, *iRORA* incomplete RPE and outer retinal atrophy, *cRORA* complete RPE and outer retinal atrophy.

## Discussion

This study prospectively investigated the movement and size of HRCs seen on AOO in non-neovascular AMD. This study adds to our knowledge of HRCs and provides a novel method of phenotyping them, which could indicate a future role for AOO in assessing HRCs as a biomarker in non-neovascular AMD.

Flood illumination adaptive optics is particularly well suited for assessing HRCs, as their high contrast hyporeflective signal makes them more apparent with flood illumination AOO than adaptive optics scanning laser ophthalmoscopy [3], which provides images of higher resolution. HRCs are thought to correspond with melanin-containing cells that appear as HRFs and RPE thickening on OCT [3], which are a common OCT finding in AMD and a robust biomarker for disease progression [13–15]. We observed many more HRCs than HRFs when correlating AOO images with OCT. Their reproducibility makes artefactual signal unlikely, and this could suggest that some HRCs are not hyperreflective on OCT, perhaps suggesting a different cell type or contents. The lower number of HRF could also be accounted for by difficulty in identification adjacent to hyperreflective structures on OCT, such as the ellipsoid zone layer (EZ) or RPE, or that HRF are missed in the gaps between slices (around 30 µm in this study).

While the majority of HRFs are agreed to represent melanin-containing cells, their cellular origin divides opinion. They have generally been interpreted as migrating RPE cells [16], but recent evidence has emerged suggesting MPs may make up part, or the majority of HRFs [6, 17, 18]. RPE thickening and HRF have significant overlap, and are thought to be a continuum of “sloughed” RPE cells in the sub-retinal space that may progress to “intraretinal” cells, or “dissociated” cells over geographic atrophy [19, 20]. Other potential origins of hyperreflective signal and HRFs include lipid-laden macrophages [21, 22] or cone lipofuscin granules [23]. These are unlikely to appear as HRCs on AOO as lipofuscin and lipids appear hyperreflective on AOO imaging [3, 24], but the appearance of melanolipofuscin on AOO is unknown.

It was outside the remit of this study to determine the cellular origin of HRCs, but the evidence gathered improves our understanding of HRCs and allows us to consider the possible candidates. The heterogeneity of HRCs in size and morphology makes it likely there are multiple structures that appear as HRCs. The size of HRCs in our sample showed a wide range, with extremely small HRCs at just under 3 µm in diameter, to bodies an order of magnitude larger (Fig. 1d). The majority of HRCs were circular or amoeboid in shape, with a 15.8 µm mean diameter, which is slightly larger than the 12.8 µm diameter of microglia found in the ganglion cell layer by Rui et al. [9]. The shape and size of HRCs in our study could be consistent with activated microglial cells within the outer retina, but could also be consistent with RPE cells, which can vary greatly in diameter [25]. HRCs in our study had a smaller mean diameter than the 19.1 µm measured by Gocho et al. [10], which could be consistent with our inclusion of images without iRORA or cRORA, where the HRCs appeared smaller. The difference in size of HRCs over drusen compared to iRORA or cRORA could be consistent with greater numbers of activated, enlarged microglia over atrophy, or a difference in cellular constituents, with smaller MPs predominating over drusen, and an increase in larger, “dissociated” RPE after progression to cRORA. In our population, we noted significant size variations of HRCs, with a less common kind appearing as particularly small, round, or amoeboid bodies around 3–10 μm in diameter (Fig. 1b). Multiple small HRCs were seen in all images, and like their larger counterparts, could appear circular or amoeboid in shape. It is unclear if these small HRCs represent a distinct origin or describe a heterogeneous size distribution of one cell line. RPE melanosomes have been measured around 2.5 µm in diameter [26], and could represent the small HRCs measured in this study. HRCs were smaller in images with relatively intact cone mosaics, and it is also possible the waveguiding or lens-like effect of the cone outer segments [27] breaks up or reduces the image of an underlying HRC. The presence of small HRCs in cRORA suggests that they are not a purely optical effect, and HRC size may therefore be a useful biomarker of disease in AMD. Medium-sized HRCs around 10–25 μm tended to be well defined, where their margins were identifiable, which may suggest they are comfortably within the FIAO camera’s depth of focus. Larger HRCs had greater diameters than could be expected for microglia and may represent enlarged macrophages or RPE cells, but correlation with OCT showed that some clusters of HRCs out of the AOO focal plane could appear as single large HRCs.

HRCs are a striking and common feature of AMD, which are known to be highly dynamic, with the majority of HRCs moving perceptibly in images taken weeks apart [10]. They have previously been described as moving up to 1 µm/day [3], but we have shown that HRCs can migrate significantly faster. No HRCs smaller than 7.9 µm were amongst the mobile HRCs measured, but conclusions cannot be drawn due to the small number of patients in this study. The rate of HRC movement is comparable, but slower than the 0.02 µm/s speed of microglia in the ganglion cell layer observed in vivo in normal human eyes by Rui et al. [9]. The fast-moving HRCs imaged in this study move at a speed that could be consistent with microglial cells, but other cellular origins, such as RPE cells, should be considered, as their migration speed is unknown. Future studies could inform our work further by measuring HRF migration speed using OCT, but may be limited by relatively reduced resolution and movement of HRCs in gaps between slices.

The strengths of this study include its use of the commercially available Rtx-1 camera, with which much evidence has been previously published, including work with HRCs. The method was effective in identifying mobile HRCs, and this was supported by the lack of detectable movement in images taken just seconds apart. The limitations of the study include the small sample size with a heterogeneous population of intermediate and late non-neovascular AMD. The use of only two imaging timepoints necessitated an assumption of linear movement of HRCs and made identification of the origin and destination of mobile HRCs uncertain. Measurements were limited by the lack of available data on axial length. Another limitation was that the methods used would only measure two-dimensional movement and are likely to have missed movement in a vertical direction.

In summary, AOO is a useful modality for assessing HRC features, including size, and analysis of sequential images can detect minute changes and measure the speed of migration. The majority of HRCs are a diameter consistent with microglia, but could also represent RPE cells. HRCs appear larger in images of cRORA, which may indicate activation of, or a change in, the constituent cells. Movement of some HRCs can be detected in as little as 49 min. Further work is required to determine if HRC diameter or motility is associated with disease activity and could be a biomarker of disease progression in non-neovascular AMD.

## Summary

### What was known before:

- Hyperreflective foci appear as hyporeflective clumps (HRC) on adaptive optics ophthalmoscopy. HRC is a common finding in age-related macular degeneration (AMD).
- HRCs are around 19 μm in diameter and are highly dynamic, moving up to 0.1 μm per day.

### What this study adds:

- HRCs can move up to 0.1 μm per second. HRC size varies, and they appear larger in images of atrophy. HRC size and speed may represent useful biomarkers in AMD.

## Supplementary information

Supplementary video 1: Overlaid adaptive optics ophthalmoscopy images taken 153 min apart. AOO images are overlaid in video to assist identification of mobile hyporeflective clumps (HRCs)(marked with white triangles and then white circles). Note that the bottom left marked HRC appears to vary in hypo-reflectivity between images. This may indicate a change in depth, so that it no longer lies entirely within the camera focal plane, or it could indicate a different, less hyporeflective HRC. If this is the case, it may have clustered with other HRCs or left the image frame, and so was not included in the movement analysis. The complete retinal pigment epithelium and outer retinal atrophy (cRORA) is partially captured and appears as a relatively homogenously hyper-reflective area on the left side of the image with overlying individual and clusters of hyporeflective HRCs. On the upper right margin of the cRORA is a highly hyper-reflective focus with overlying HRCs. This corresponds with hyper-reflective material on Bruch’s membrane on optical coherence tomography B scan (not shown). The retinal vessels can be observed rising inferiorly through the centre of the image and assist with alignment of image series.

## Data Availability

The datasets generated during and/or analysed during the current study are available from the corresponding author on reasonable request for ethics-approved projects.
